# Artificial intelligence for biodiversity and tourism governance: predictive insights from multilayer perceptron models in Amazonia

**DOI:** 10.3389/frai.2026.1702544

**Published:** 2026-02-05

**Authors:** Jessie Bravo-Jaico, Oscar Serquén, Roger Alarcón, Juan Eduardo Suarez-Rivadeneira, Wilfredo Ruiz-Camacho, Freddy A. Manayay

**Affiliations:** 1 Digital Transformation Research Group, National University Pedro Ruiz Gallo, Lambayeque, Peru; 2 Faculty of Systems Engineering and Electrical Mechanics, National University Toribio Rodríguez of Mendoza, Bagua, Peru; 3 Biosystems Engineering Professional School, National University Toribio Rodríguez of Mendoza, Chachapoyas, Peru

**Keywords:** biodiversity conservation, multilayer perceptron neural networks, Peruvian Amazon, sustainable tourism, tourism demand forecasting

## Abstract

Tourism in biodiversity-rich regions was among the sectors most severely disrupted by the COVID-19 pandemic, which amplified existing socioeconomic vulnerabilities and placed cultural and natural heritage conservation at risk. In the Peruvian Amazon, Bagua Province illustrates this challenge, where experiential tourism is central to local livelihoods yet lacks adaptive management tools to support a sustainable recovery. To address this gap, this study introduces an integrated approach that combines artificial intelligence with biodiversity conservation through the application of multilayer perceptron (MLP) neural networks. By analyzing two decades of domestic visitor data (2003–2023), the research explores how predictive modeling can inform tourism governance in fragile ecosystems. Two scenarios were evaluated: one incorporating the complete dataset and another excluding the anomalous year 2020, heavily disrupted by the pandemic. The findings show that MLP models are capable of capturing visitor dynamics and forecasting demand fluctuations with notable accuracy. This predictive capacity allows for more adaptive planning of ecologically sensitive sites, such as the Tsunsuntsa Waterfall, where balancing visitor inflows with ecological thresholds is essential to preventing overtourism. Beyond technical accuracy, the study highlights the strategic potential of artificial intelligence as a governance tool that strengthens resilience in post-pandemic contexts, offering actionable insights for harmonizing socioeconomic recovery with biodiversity preservation. By positioning neural networks as vital instruments for sustainable destination management, this research contributes a reproducible model that can be adapted to other vulnerable regions worldwide. It underscores the value of integrating advanced computational methods into tourism governance frameworks, ultimately bridging technology and conservation to foster long-term sustainability.

## 1 Introduction

The tourism sector, over the last decades, has emerged as a fundamental pillar of the global economy, playing a crucial role in the socioeconomic development of nations (Zheng and Zhang, 2023). However, the recent COVID-19 pandemic has generated an unprecedented crisis in this area, negatively impacting tourism activity worldwide (Andariesta and Wasesa, 2022; Nguyen-Da et al., 2023). According to studies by the World Tourism Organization (UNWTO), in 2020, international tourism recorded a 74% drop compared to the previous year, representing the largest contraction in the history of the sector (Priyamal and Rupasingha, 2023; Talwar et al., 2022).

In Peru, the situation was somewhat similar. Despite having vast natural, cultural and historical resources, tourism experienced a significant decline (Gago et al., 2022; Menchero Sánchez, 2020), seriously affecting the economy and welfare of communities dependent on this activity (Meza, 2020; Paredes Izquierdo et al., 2020). According to data from the National Institute of Statistics and Informatics (INEI), in 2020, the arrival of foreign tourists was reduced by 94.9% compared to the previous year, evidencing the devastating impact of the pandemic on the tourism sector (Paredes Izquierdo et al., 2020).

The same was observed in the province of Bagua (Amazonas Region), which suffered a more pronounced impact, given that the province of Bagua is known for its experiential tourism, folklore, natural sites and ethnology, activities that were restricted during the pandemic. These activities were restricted during the pandemic, putting economic stress on tourism businesses and the families that depended on this activity. This represents significant challenges in terms of tourism development and management for Bagua (Cueva Vega and Rojas Vin, 2023; Neyra Paredes et al., 2022), which added to the lack of adequate infrastructure, limited tourism promotion and scarce financial resources, hinder the growth of tourism in the province.

In this context, the promotion of tourism in the province of Bagua represents an urgent priority, not only from an economic perspective, but also from a social and cultural one (Suárez Rivadeneira et al., 2025). The revitalization of this activity will not only contribute to the generation of employment and increased income in the region, but will also promote the preservation of cultural identity, the strengthening of intercultural relations and the appreciation of biodiversity. An approach this problem and its underlying factors would strengthen tourism and, consequently, raise awareness among the local population about caring for the province’s biodiversity (Cueva Vega and Rojas Vin, 2023).

In addition to the above, the lack of investment in tourism infrastructure, the absence of effective public policies and the poor training of personnel in the sector have contributed to the current situation (Llanos Flores et al., 2021). Originating a decrease in economic income, increased unemployment and loss of development opportunities, negatively affecting the local population and the sustainability of the tourist destination in the region (Tudela et al., 2022).

Against this backdrop, the need to adopt innovative approaches and predictive tools to improve tourism management and decision making is evident. In this sense, neural networks are presented as a powerful and effective tool to analyze complex data, identify patterns and trends, and predict the behavior of tourism management indicators, also the results also serve as a basis for infrastructure planning, which is used to minimize the ecological footprint (Danbatta and Varol, 2022; De Jesus and Samonte, 2023; Huang and Zhai, 2023; Tong and Ruan, 2023). Therefore, the purpose of this research is to use neural networks for post-pandemic sustainable management of tourism management indicators in the province of Bagua, Amazonas. Through the analysis of historical data and the application of predictive models, we seek to provide valuable insights that contribute to the formulation of effective strategies and policies for the reactivation of tourism in the region.

## 2 Literature review

Contemporary research demonstrates a growing reliance on artificial intelligence for tourism forecasting, with neural networks emerging as a predominant technique for demand prediction across diverse geographical contexts (Table 1).

**Table 1:** State of the art in AI applications for sustainable tourism management.

Authors/Year	AI technique(s) employed	Predicted indicators	Geographical context	Key findings	Identified gaps
Nguyen-Da et al. (2023)	Hybrid CNN-LSTM	Post-COVID-19 tourism demand	Vietnam	Captures non-linear patterns during disruptions (MAPE: 4.7%)	Neglects ecological carrying capacity considerations
Bozkurt and Şeker (2023)	Convolutional Neural Networks	Tourism pressure at UNESCO sites	Global (32 heritage sites)	Predicts visitor-degradation correlation (AUC: 0.89)	Not adapted to Amazonian contexts
Huang and Zhai (2023)	ANN with NSGA-II optimisation	Sustainable rural economic development	Rural China	Integrates ecological-economic indicators (MSE: 0.021)	Lacks data on indigenous communities
García-García et al. (2023)	MLP with cross-validation	Cultural tourist profiles	Córdoba, Spain	Enables heritage-based segmentation (F1-score: 0.87)	No models for fragile biodiversity contexts
De Jesus and Samonte (2023)	Artificial Neural Networks (ANN)	Tourism Demand	Philippines	ANNs outperform traditional methods (R-squared value: 0.926 and MAPE 13.9%)	Need for local socio-economic variable integration
Bravo et al. (2023)	Multilayer Perceptron (MLP)	Tourism Demand (National Visitors)	Perú	linear regression model. Best algorithm to prediction of national and foreign tourists (R-squared value: 0.88 and 0.76)	No linkage to sustainability metrics
Andariesta and Wasesa (2022)	MLP, Random Forest, Gradient Boosting	International tourist arrivals	Indonesia	Hybrid models with multisource data improve crisis-period accuracy (RMSE: 0.18)	Limited integration of environmental variables
Danbatta and Varol (2022)	ANN-Polynomial-Fourier	Seasonal tourist flow	Nigeria	Hybrid models outperform ARIMA in accuracy (R^2^: 0.94)	Omits biodiversity impact assessments
Talwar et al. (2022)	ANN with personality analysis	Post-COVID-19 travel intention	India, Japan, Saudi Arabia	Psychological variables enhance accuracy (Accuracy: 91.4%)	No linkage to sustainability metrics
Afrianto and Wasesa (2022)	Tree-Based Models & Quarantine Search	Tourism Demand	Indonesia	Historical data and search queries significantly influence accuracy (MAPE 39.6%)	Validation gaps in low-digital-connectivity regions
Ramos-Carrasco et al. (2019)	Multilayer Perceptron (MLP)	Domestic tourism demand	Peru (coastal regions)	Robust validation in volatile environments (MAE: 0.089)	Does not address post-pandemic effects

Studies by Nguyen-Da et al. (2023) in Vietnam and De Jesus and Samonte (2023) in the Philippines have validated hybrid CNN-LSTM and ANN architectures for capturing non-linear disruptions in post-COVID tourism flows, achieving MAPE values of 4.7 and 13.9%, respectively. Similarly, Andariesta and Wasesa (2022) and Ramos-Carrasco et al. (2019) confirmed the robustness of MLP models in volatile environments (RMSE: 0.18; MAE: 0.089) across Indonesia and coastal Peru.

However, critical gaps persist in the literature: (1) Neglect of ecological variables, evidenced by Bozkurt and Şeker (2023) global UNESCO study omitting Amazonian carrying capacity metrics; (2) Insufficient integration of biodiversity considerations, with Huang and Zhai (2023) acknowledging data deficits regarding indigenous communities; and (3) Post-pandemic sustainability blind spots, as García-García et al. (2023) identified no models for fragile ecosystems. While Afrianto and Wasesa (2022) revealed the influence of digital connectivity on prediction accuracy (MAPE 39.6%), and Talwar et al. (2022) achieved 91.4% accuracy in behavioural forecasting, none bridged AI-driven demand prediction with biodiversity conservation, a gap this study addresses through MLP modelling in Bagua’s hyper-diverse Amazonian context, integrating ecological sensitivity metrics absent in prior works like (Bravo et al., 2023).

## 3 Theoretical framework

The province of Bagua is located on the northern platform of the lower Utcubamba valley, at an altitude ranging from 400 to 575 meters above sea level, it is crossed by the Chiriaco and Utcubamba rivers, and the Atunmayo, Capallín, Keta, Amojau, and other streams. It has a wide variety of tourist attractions, as shown in Table 2, highlighting the varied biodiversity, cultural manifestations and natural sites (Dirección SubRegional Comercio Exterior y Turismo 2019). The promotion of tourism activity in the province of Bagua - Amazonas is of crucial importance for the socioeconomic development of the region, given that this activity will not only boost the local economy, but also promote the preservation of the cultural and natural heritage of the area. In this context, management indicators play a fundamental role in providing key information for strategic decision-making in the tourism sector.

**Table 2:** Tourist places in the province of Bagua, sectors and attractions.

District	Place	Name of the resource	Principal attraction
Copallin	Cambio Pitec	Cambio Pitec Caverns	Cultural manifestations
	Sector soles	Los Soles Caverns	Cultural manifestations
	San José Alto	Natural Waterfalls	Natural site
	Cambio Pitec	Tiger Falls	Natural site
	Cambio Pitec	El Tigre Archaeological Site	Cultural manifestations
La Peca	Sector del Arenal	Arenal Canyon	Natural site
	Caserío Arrayán	Llactan Archaeological Site	Cultural manifestations
Bagua	Caserío Casual	Casual Archaeological Site	Cultural manifestations
	Caserío las Juntas	Los Peroles and Las Juntas Archaeological site	Cultural manifestations
	Sector Dos de mayo	Cruz del Conjuro Tourist Site	Cultural manifestations
	Bagua	Main square	Cultural manifestations
	Bagua	Bagua Mother Church	Cultural manifestations
	Bagua	Utcubamba River	Natural site
	Rentema	“Pongo de Rentema”	Natural site
	Bagua	Grano de oro	Folklore
	Bagua	Guerreros de Sambajai	Folklore
	Rentema	Valley of the Dinosaurs	Natural site
El Parco	El Parco	Craft Manufacturing	Folklore
Aramango	Nueva Esperanza	Numparket Waterfall	Natural site
	Tsunsuntsa	Tsunsuntsa Waterfall	Natural site
	El Porvenir	The Porvenir Lagoon	Natural site
Imaza	Native Communities	Masato de Yuca (drink)	Folklore
	Various Communities	Imaza y Aramango	Ethnology
	Chiriaco	Chiriaco River	Natural site
	Native Communities	Craft Manufacturing	Folklore
	Native Communities	Dances	Folklore
	Chiriaco- Utcubamba	Colan Mountain Range	Natural site

### 3.1 Neural networks and their application in tourism

Neural networks are computational tools inspired by the functioning of the human brain, capable of learning complex patterns from data (Cho et al., 2014; Lou et al., 2020). In the tourism domain, neural networks have been successfully used to predict key indicators, such as tourism demand, visitor behavior patterns and hotel occupancy. Previous studies have shown that these techniques can significantly improve the accuracy of predictions and provide valuable insights for tourism planning and management (Ramos-Carrasco et al., 2019).

In the context of tourism, neural networks have been successfully applied in several areas, including tourism demand prediction (De Jesus and Samonte, 2023), market segmentation (Laaroussi et al., 2023), service personalization (Kontogianni et al., 2022), price optimization, destination recommendation, and customer experience management (Hu, 2021; Ni et al., 2021). Facilitating tourism managers to anticipate fluctuations in demand (Xu and Wang, 2022), adapt their personalized marketing strategies (Gadri et al., 2021), personalize tourism service offerings (Lou et al., 2020), dynamically adjust prices according to demand, and recommend personalized tourism destinations to users (Baldigara, 2022; Talwar et al., 2022), respectively.

### 3.2 Tourism management indicators

Management indicators in tourism are metrics used to measure performance and evaluate the success of tourism initiatives (Bravo et al., 2023). Examples of these indicators include hotel occupancy, visitor flow, customer satisfaction, tourism expenditure, tourism competitiveness index, and tourist return rate (Bozkurt and Şeker, 2023; Mohamed et al., 2023; Ozturk et al., 2023). These indicators are fundamental to understand the dynamics of the tourism sector and to guide strategic decision making for the reactivation of tourism in the province of Bagua - Amazonas.

### 3.3 Predictive applications in tourism for management indicators

Previous research has explored the use of predictive models, including neural networks, to forecast management indicators in tourism turismo (García-García et al., 2023; Ni et al., 2021; Wang et al., 2023). These studies have shown that predictive tools can provide accurate and timely forecasts, allowing tourism managers to anticipate trends, identify areas of opportunity and mitigate risks (García-García et al., 2023; Tong and Ruan, 2023). It allows forecasting future hotel occupancy, identifying demand patterns and adjusting pricing and promotion strategies, as well as allowing tourism managers to predict the flow of visitors in a given period of time, and to adapt their marketing and promotion strategies (Danbatta and Varol, 2022; Huang and Zhai, 2023; Jatmika et al., 2024; Tong and Ruan, 2023). In a similar way, MLP models allow for quick changes to deal with crises and make systems stronger after a pandemic (Andariesta and Wasesa, 2022).

### 3.4 Initiatives for tourism reactivation in similar regions

The analysis of tourism reactivation initiatives in regions with similar characteristics to the province of Bagua - Amazonas offers valuable insights for the design of effective strategies. Case studies from other regions have shown how the accurate prediction of management indicators would have contributed to the successful planning and execution of tourism reactivation projects (Gago et al., 2022; Llanos Flores et al., 2021).

In conclusion, the application of neural networks for the prediction of management indicators in tourism offers a unique opportunity to improve tourism planning and management in the province of Bagua - Amazonas. However, there are still gaps in the literature that require further research, especially in terms of adapting predictive models to specific contexts and integrating local data. It is recommended that future research focus on addressing these areas to strengthen predictive capacity and improve the effectiveness of tourism reactivation strategies in the region.

## 4 Methodology

**Figure 1:**
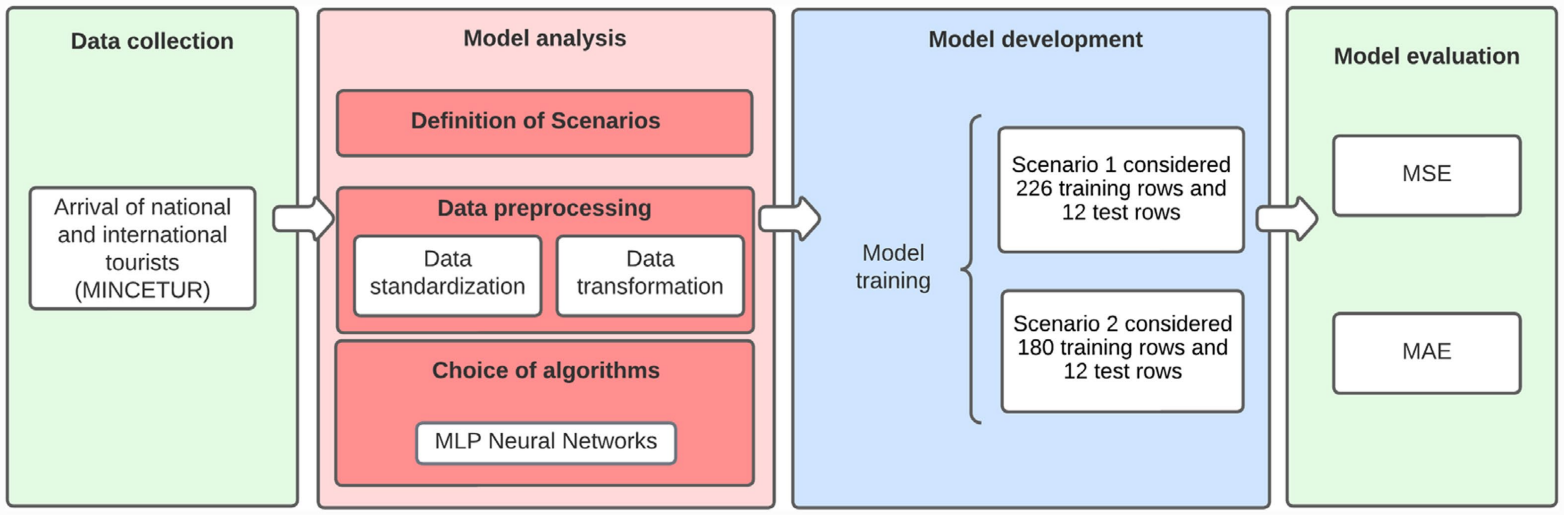
Methodology of applied research.

The methodology used in this research work was structured in the following steps, as shown in Figure 1.

### 4.1 Data collection

Data on arrivals of both national and foreign tourists to the city of Bagua were obtained from the Ministry of Foreign Trade and Tourism (MINCETUR, 2024), which are freely available without copyright restrictions.

The data obtained covers the period from 2003 to 2023 for domestic visitors. This information was extracted from Excel files and integrated into a single data source for further processing. It was determined that due to COVID-19, very low values were recorded from March 2020 to January 2021, after which the numbers returned to average values.

**Figure 2:**
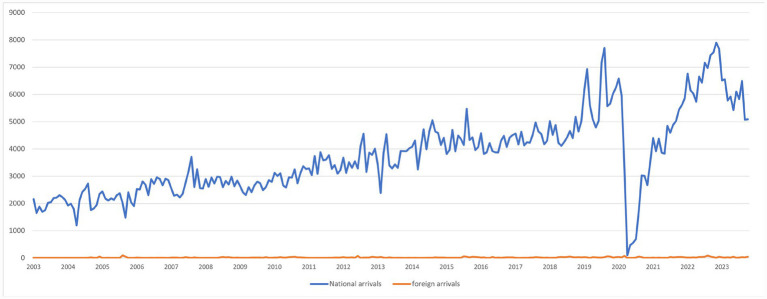
National and foreign arrivals data.

An analysis of the data was conducted, concluding that only national visitor data from January 2003 to October 2023 should be considered, due to their significant variation and increasing trend. The data for international visitors remained constant and with minimal values (see Figure 2).

Table 3 shows the variables used in the study. The scenarios used are characterized by being time series, so the visitor’s variable serves as both a predictor variable and a variable to be predicted.

**Table 3:** Variables used in the model prediction.

Variable	Variable name	Description	Source
Predicted Variable	visitors_{t1} al visitors_{t12}	Number of visitors in months t1 to t12	Prediction
Predictor Variables	visitors_{t-1}	Number of visitors in the previous month	MINCETUR (2024)
	visitors_{t-204}	Number of visitors in the previous month 204	MINCETUR (2024)
	date	Time variable used to extract seasonal components (month, year)	MINCETUR (2024)

### 4.2 Scenario definition

Two analysis scenarios were defined:

**Scenario 1:** Considered all data collected from January 2003 through October 2023.

**Scenario 2:** Excluded 2020 data due to immobility caused by the COVID-19 pandemic. In this scenario, 2020 data were predicted using data from previous years.

### 4.3 Data preprocessing

The data were scaled to a range between −1 and 1 to optimize the performance of the neural networks.

The data were transformed from time series format (one column with 250 rows) to a 13-column, 238-row format for the first scenario. For the second scenario, the data were transformed from one column with 204 rows to 13 columns and 192 rows.

### 4.4 Data splitting

**Figure 3:**
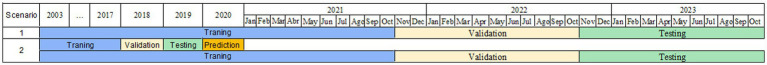
Dataset partition: training, validation, and test.

The data were divided into training, validation, and test sets for both scenarios. For scenario 1, 226 training rows, 12 validation rows, and 12 test rows were considered, while for scenario 2, the prediction for the year 2020 was first made, considering 180 training rows, 12 validation rows, and 12 test rows. Finally, the prediction for the year 2020 was included for the partition in this scenario, as shown in Figure 3.

#### 4.5 Model development

A multilayer perceptron (MLP) type neural network was defined for training and prediction.

In a neural network, the arrangements of neurons form clusters or layers, at a distance from the input and output layers of the network. Thus, the parameters of neural networks can be described as the number of layers, the degree of connectivity and the type of connection between neurons (Montesinos López et al., 2022). These can be classified into monolayer and multilayer networks, the latter being the one that will be used for the development of this research.

#### 4.5.1 Technical specifications of the feedforward model

The model implemented corresponds to a Feedforward (Sequential) Artificial Neural Network oriented towards regression tasks. The architecture follows a unidirectional data flow and is structured into three main components: input/hidden layer, flattening layer, and output layer (Bergmeir et al., 2018; Tashman, 2000).

The input/hidden layer consists of a dense (fully connected) layer configured to receive an input tensor with shape (1, 12), corresponding to the time window or set of features used. This layer contains 12 neurons activated by the tanh function, which normalizes their outputs in the interval [−1, 1]. The use of this activation allows nonlinear relationships to be captured and negative values present in the time series to be handled.

The architecture then includes a flattening layer, whose purpose is to transform the multidimensional tensor produced by the hidden layer into a one-dimensional vector. This process allows the internal representation of the model to be properly connected to the final layer responsible for prediction.

Finally, the output layer consists of a dense layer with a single neuron, designed to produce a scalar output corresponding to the estimated value. This layer also uses the tanh activation function, which means that the predictions generated by the model remain restricted to the interval defined by that activation, facilitating numerical stability and interpretation of the output range.

#### 4.5.2 Summary of hyperparameters and configuration

The Adam optimizer was used to compile the model, selected for its ability to adjust parameters using adaptive estimates of first- and second-order moments, which promotes stable convergence in regression tasks.

The loss function used was Mean Absolute Error (MAE), with the aim of minimizing the average difference in absolute terms between the predicted and observed values, thus providing a robust measure against outliers.

During the training process, the model’s performance was evaluated using the MSE (Mean Squared Error) and MAE metrics, which allowed for monitoring both the average magnitude of errors and sensitivity to large deviations, ensuring a comprehensive evaluation of predictive behavior.

#### 4.5.3 Training specification and adjustment

The supervised learning phase uses the fit method, adjusting the synaptic weights of the neural network to minimize the previously defined loss function. The process has the following parameters:

- Training Data: The model is fed with the tensors x_t (features) and y_t (targets), which represent the main learning dataset.
- Iteration Cycles (Epochs): A total of 30 epochs has been set (EPOCHS = 30). This means that the learning algorithm will go through the entire training dataset 30 times to refine the model parameters.
- Batch Size: The batch size has been dynamically set equal to the PASOS variable. This determines that the gradient update (Backpropagation) will occur every time 12 samples are processed.
- Validation Strategy: Hold-out cross-validation is implemented in each epoch, using the x_v and y_v sets. This allows the model’s performance to be monitored on unseen data in real time, facilitating early detection of overfitting.

A learning rate of 0.001 was established, the random seed was not defined, no L1 or L2 regularization penalties were applied in the dense layers, which are defined only by the ‘tanh’ activation function and the number of neurons.

Python version 3.11 was used as the software framework, and Keras was applied as a high-level API to build and train the neural network model (Sequential, Dense, Activation, Flatten, Dropout). For machine learning and preprocessing, Scikit-learn (sklearn) was used, specifically StandardScaler, MinMaxScaler, and OneHotEncoder, and a regression model (MLPRegressor) was also imported.

For data manipulation and analysis, Pandas and NumPy were used for numerical operations and matrix handling, and finally, Matplotlib was used for graph visualization.

The training of the model was performed using the designated training data.

### 4.6 Model evaluation

The model was evaluated with the test data, using indicators such as mean squared error (MSE) and mean absolute error (MAE).

### 4.7 Prediction

Scenario 1: Prediction was performed for the last 12 months, from November 2023 to October 2024.

Scenario 2: First, the 2020 data were predicted and replaced with the actual data. Then, the prediction was performed for the last 12 months, from November 2023 to October 2024, as in scenario 1.

## 5 Results

**Figure 4:**
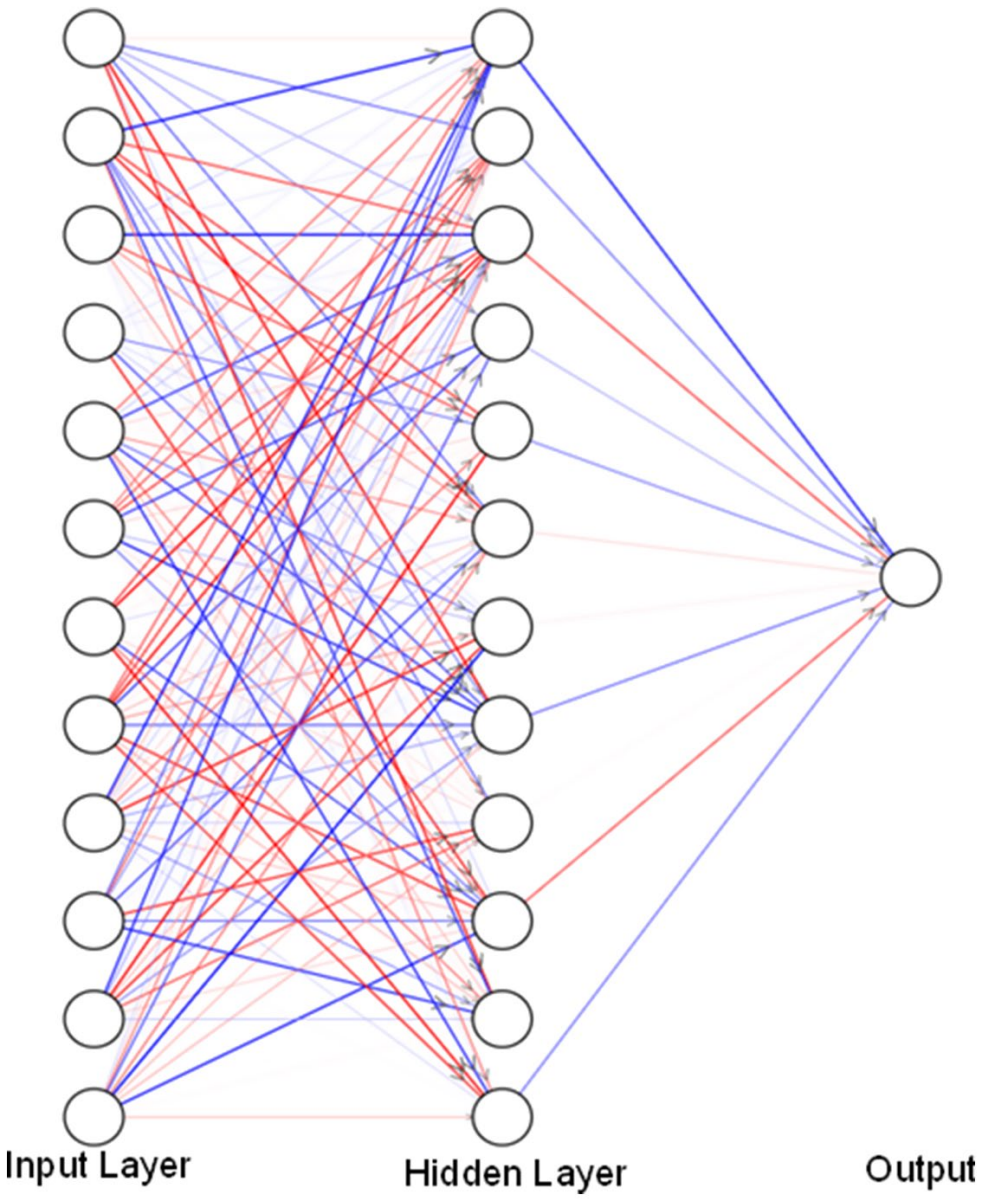
Neural network model applied.

The data obtained were for the period from January 2003 to October 2023. After consolidating the data, the data were prepared for input to the neural network, the data were scaled to values between −1 and 1, which allow the neural networks to work optimally. The model used, see Figure 4, consists of an input layer of 12 neurons representing a block of 12 months, then a hidden layer is included that connects with the input, and finally an output layer that generates the prediction of a single month. This process is repetitive taking the generated output as a new input to the neural network.

The application of the model was performed in two scenarios. The first one considering all the data obtained during January 2003 and October 2023, the second scenario did not use the 2020 data because the values were considered outliers due to the pandemic to avoid predicting unexpected results, to avoid this, the prediction of 2020 was made taking into account the data from January 2003 to December 2019, finally the data from January 2021 to October 2023 was added.

Table 4 shows the above, including the forecast in both scenarios for the subsequent 12 months from November 2023 to October 2024.

**Table 4:** Scenario 1 and scenario 2 with respect to the manipulated data.

Data category	Date	Visitors	Data category	Date	Visitors
Real data	January - 2003	2,151	Real data	January - 2003	2,151
	February - 2003	1,651		February - 2003	1,651
	...	...		...	...
Visitors in pandemic year	January - 2020	6,616	Visitor prediction for the year 2020	January - 2020	4,685
	February - 2020	5,979		February - 2020	5,048
	March - 2020	3,292		March - 2020	5,698
	April - 2020	102		April - 2020	5,316
	May - 2020	469		May - 2020	5,340
	June - 2020	552		June - 2020	4,998
	July - 2020	710		July - 2020	5,173
	Aug - 2020	1760		Aug - 2020	6,003
	September - 2020	3,050		September - 2020	6,449
	October - 2020	3,010		October - 2020	6,102
	November - 2020	2,766		November - 2020	5,864
	December - 2020	3,508		December - 2020	5,590
Real data	...	...	Real data	...	...
	September - 2023	5,090		September - 2023	5,090
	October - 2023	5,124		October - 2023	5,124
Prediction	November - 2023	6,857	Prediction	November - 2023	4,897
	December - 2023	6,971		December - 2023	4,961
	January - 2024	7,011		January - 2024	5,633
	February - 2024	6,701		February - 2024	6,301
	March - 2024	6,733		March - 2024	5,442
	April - 2024	6,083		April - 2024	5,431
	May - 2024	6,125		May - 2024	5,452
	June - 2024	5,468		June - 2024	6,074
	July - 2024	5,754		July - 2024	6,543
	August - 2024	5,317		August - 2024	6,713
	September - 2024	5,894		September - 2024	5,333
	October - 2024	5,395		October - 2024	5,654
Scenario 1.- Visitor prediction from November 2023 to October 2024				Scenario 2.- Visitor prediction for the year 2020 and from November 2023 to October 2024	

In both cases, the same neural network model was applied, and the following results were obtained, as shown in Table 5, which shows that the mean square error (MSE) indicator in scenario 2 offers better results than in scenario 1. On the other hand, the evaluation with the mean absolute error (MAE) indicator is the same, with the results in scenario 2 being better than in scenario 1.

**Table 5:** Comparison of the two scenarios.

Indicators scenario 1		Indicators scenario 2	
MSE	MAE	MSE	MAE
0.023	0.101	0.014	0.092

**Figure 5:**
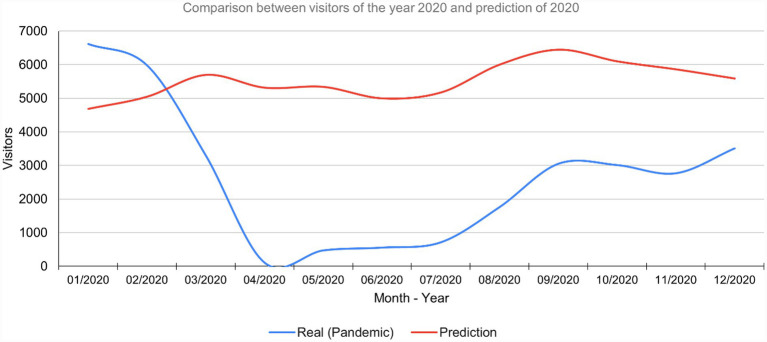
Comparison of visitor scenarios for the year 2020.

After making the prediction for the year 2020 using the neural network model, it is evident that it shows the expected trend of visitors in that year, which was abruptly modified by the effects of the pandemic. Figure 5 shows how the red line follows the expected sequence while the blue line shows the abrupt drop in March 2020, when the pandemic was officially declared in Peru.

**Figure 6:**
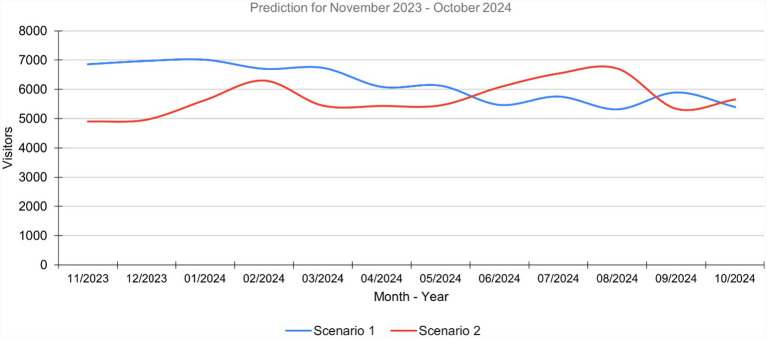
Comparison of the prediction of the two scenarios between actual values and predicted values.

As described at the beginning of this section, a prediction was made for the last 12 months, using both scenarios. Figure 6 shows the results and compares them graphically, and although there are differences in the curves, the results of the MSE and MAE indicators show better results in scenario 2.

**Figure 7:**
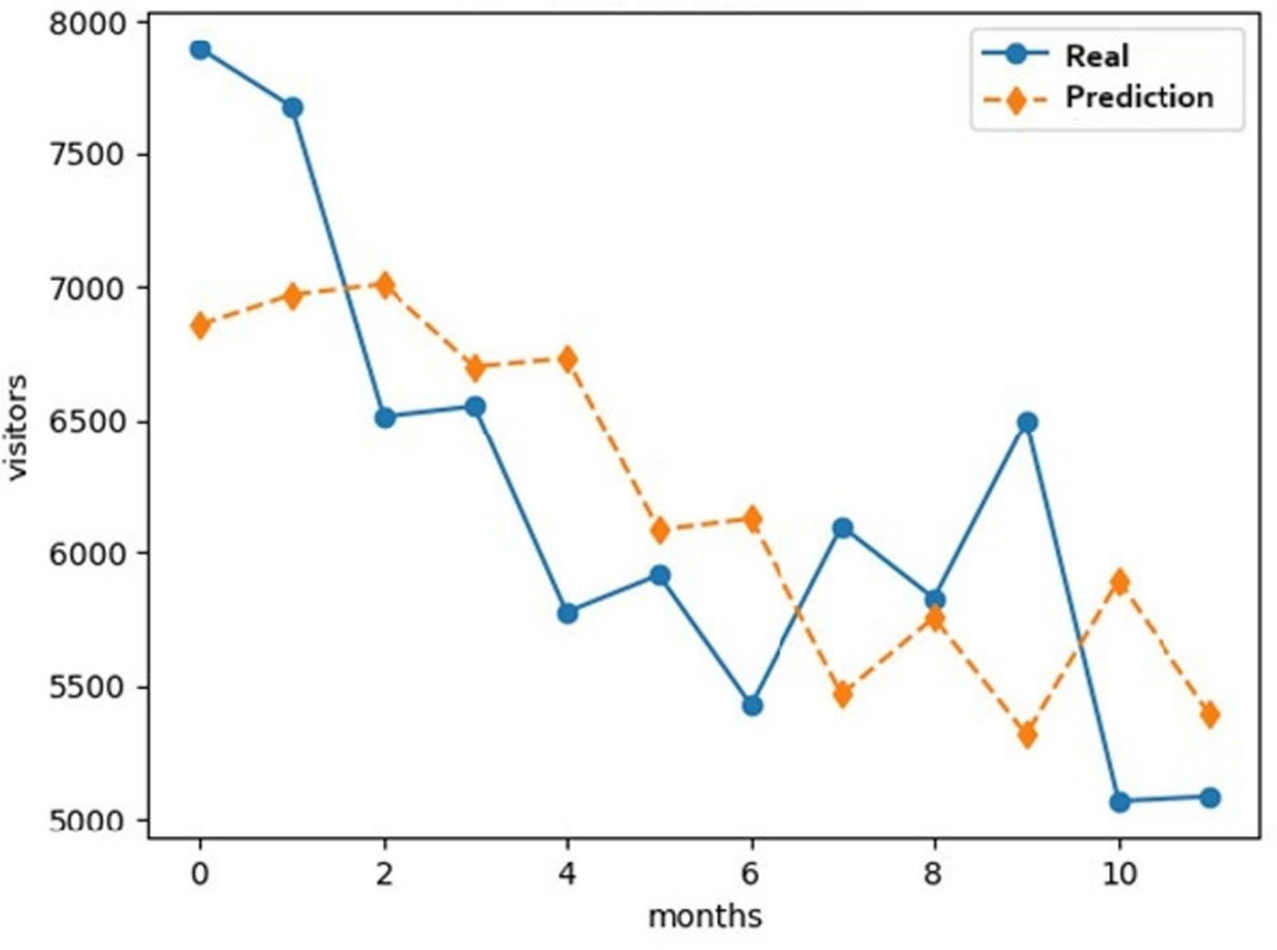
Comparison between prediction and actual data.

Figure 7 shows the actual data and 12-month data predicted by the neural network model for scenario 1.

**Figure 8:**
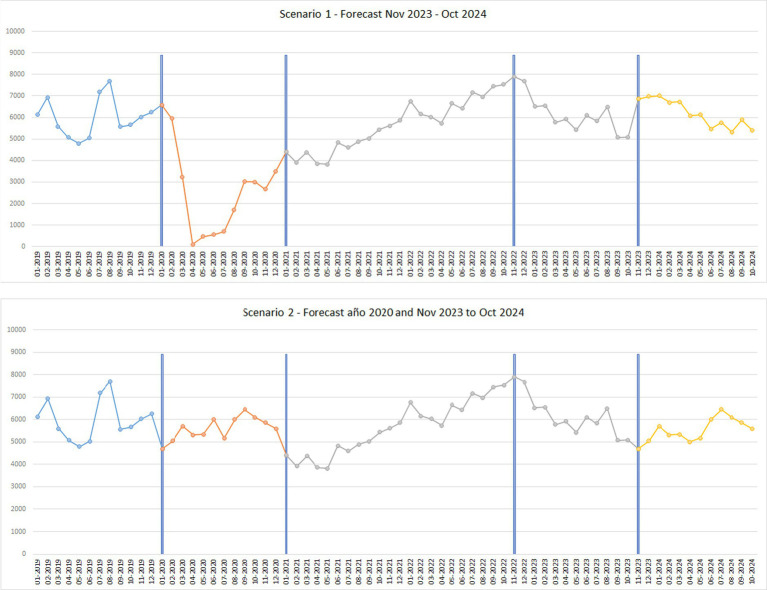
Predictions in both scenarios.

Figure 8 shows the forecast of domestic visitors, showing that the trend of visitors remains stable with a tendency to increase. In addition, it can be observed that in July it maintains a high value, while in the rest of the months it fluctuates up and down. It should be noted that scenario 1 shows a constant drop in these values, while scenario 2 shows an upward trend.

**Figure 9:**
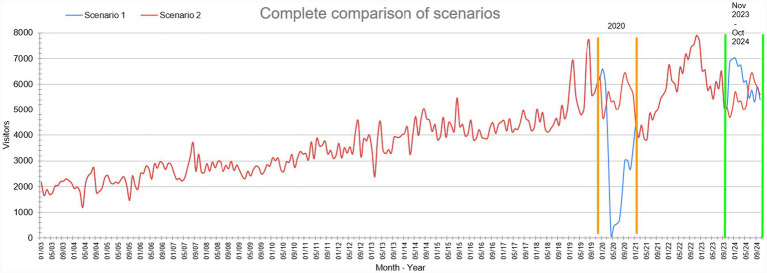
Comparison of all data from January 2003 to October 2024 for both scenarios.

Figure 9 shows the complete comparison of scenarios from January 2003 to October 2024. The orange stripes highlight the year 2020 with the actual data in this case scenario 1 and the data predicted with scenario 2, and the green stripes show the final results of the prediction from November 2023 to October 2024.

## 6 Discussion

Tourism activity in the province of Bagua - Amazonas is crucial for the socioeconomic development of the region. The results obtained in this research confirm the usefulness of neural networks to predict tourism management indicators, providing valuable information for strategic decision making in the sector.

The results show that the neural network model used, specifically the multilayer perceptron (MLP), has successfully predicted the tourism management indicators, with better results in scenario 2 (excluding the 2020 outlier data due to the COVID-19 pandemic). This is evidenced by the lower mean squared error (MSE) and mean absolute error (MAE) values in scenario 2, suggesting that the exclusion of outliers improves the accuracy of the predictions.

These findings are in line with previous studies demonstrating the ability of neural networks to handle complex data and provide accurate predictions in the tourism sector (Ramos-Carrasco et al., 2019). The ability of neural networks to adapt and learn from historical data allows tourism managers to anticipate trends and make informed decisions (Gadri et al., 2021).

Management indicators are essential to measure the performance of the tourism sector and to evaluate the success of revival strategies (Bravo et al., 2023). In this study, the prediction of domestic visitors showed an increasing trend, especially notable in specific months such as July. This type of information is crucial for tourism managers, as it allows them to adjust marketing and promotion strategies according to demand expectations (De Jesus and Samonte, 2023; Nguyen-Da et al., 2023).

The prediction accuracy of these indicators also facilitates the planning of infrastructure and services, optimizing the visitor experience and improving the competitiveness of the destination (Bozkurt and Şeker, 2023; Mohamed et al., 2023). In a similar manner, predictions obtained with the aid of machine learning algorithms will allow for dynamic adaptation to crises and improvement in resilience following a pandemic (Andariesta and Wasesa, 2022). Furthermore, such models will be able to guide the management of visitor flow in fragile ecosystems (for example, the Utcubamba river basin) in order to avoid over-tourism (Andariesta and Wasesa, 2022; García-García et al., 2023).

The comparison between the two scenarios used in this research highlights the importance of the quality and relevance of historical data in the prediction of tourism indicators. The exclusion of 2020 outlier data resulted in more accurate and consistent predictions, suggesting that outliers can distort the results of predictive models (Danbatta and Varol, 2022).

The forecasts generated by the MLP support adaptive planning by anticipating periods of high visitor numbers at ecologically sensitive sites (e.g., Tsunsuntsa Waterfall). These results can be integrated with environmental management frameworks (carrying capacity/acceptable change limits) when thresholds are defined by ecological evidence and official guidelines. The results of this research can be used by tourism managers to anticipate fluctuations in demand, optimize prices and promotions, and improve the supply of personalized services (Baldigara, 2022; Lou et al., 2020; Talwar et al., 2022).

Despite promising results, this research has certain limitations. The exclusion of international data due to its constancy and low values may have limited the full understanding of the tourism landscape in the region. Future research could explore the integration of international data and other external factors that could influence tourism in Bagua.

In addition, the adaptation of predictive models to specific contexts and the integration of local data, specifically at the level of each tourism site, remain areas that require further research (García-García et al., 2023; Tong and Ruan, 2023). It is recommended that future research focus on these areas to strengthen predictive capacity and improve the effectiveness of tourism reactivation strategies in the region.

## 7 Conclusion

The present research has demonstrated that the use of neural networks, specifically the multilayer perceptron model (MLP), is viable and effective for predicting visitor numbers in the province of Bagua - Amazonas. Through the analysis of historical data of national visitors from January 2003 to October 2023, predictions were obtained that are necessary for tourism planning and management in the region.

The results obtained show that the MLP neural network model provides accurate predictions, especially when 2020 outliers are excluded. The lower values of MSE and MAE in scenario 2 validate the effectiveness of this approach.

Management indicators, such as visitor flow, are crucial for strategic decision making in the tourism sector. The predictions obtained can help tourism managers anticipate fluctuations in demand, optimize marketing strategies and improve service offerings.

The application of neural networks in predicting domestic visitors offers multiple practical benefits: tourism managers can use the predictions to better plan infrastructure and services, optimizing the visitor experience and improving the competitiveness of the destination; the ability to anticipate demand allows them to adjust marketing and promotional strategies more effectively, attracting more visitors and increasing tourism revenues; and the reactivation of tourism, based on accurate data, can contribute to the sustainable socioeconomic development of the region, preserving its cultural and natural heritage.

Future research should consider the inclusion of international visitor data to obtain a more complete picture of the tourism landscape in the region. It is also necessary to continue exploring the adaptation of predictive models to specific contexts and the integration of local data to improve the predictive capacity and effectiveness of tourism reactivation strategies, and to investigate other artificial intelligence and machine learning techniques, such as deep learning models, which can offer new perspectives and improvements in the accuracy of predictions.

In addition, the application of neural networks for the prediction of tourism management indicators in the province of Bagua - Amazonas represents a valuable tool for the reactivation and sustainable development of tourism in the region. The capacity of these technologies to provide accurate and timely predictions has the potential to transform the manner in which tourism is planned and managed, thereby contributing significantly to the economic and social welfare of the local community.

### 7.1 Limitations

The lack of data granularity did not allow for a deeper analysis of the relationship between monthly visitor forecasts and ecological carrying capacity thresholds, as only aggregated monthly data was available, making it impossible to identify daily peaks and link them to more detailed ecological evidence. This should be addressed in future research by incorporating daily data or data with higher temporal resolution.

This research did not incorporate formal stationarity tests or an exhaustive analysis of model residuals. In particular, statistical tests of stationarity (such as ADF or KPSS), ACF and PACF correlograms of the residuals, Ljung–Box tests, and graphs of residuals against predictions to assess the presence of autocorrelation, heteroscedasticity, or systematic biases were not included. This absence limits the ability to verify whether the residuals behave as white noise and, therefore, to fully assess the adequacy of the model. Future work should integrate these diagnostics to strengthen the validity and robustness of the results.

The research did not include comparisons with reference models, which limits the ability to contextualize the performance of the proposed model against other alternatives. Future research should incorporate comparative models to strengthen the evaluation and validity of the results obtained.

The research does not present the evaluation or calibration by forecast horizon or by month. This absence prevents the identification of seasonal biases and the evaluation of the model’s behavior over different time horizons. It is suggested that future research incorporate multi-horizon forecasting strategies and monthly analyses to obtain a more complete understanding of the model’s performance.

## Data Availability

The raw data supporting the conclusions of this article will be made available by the authors, without undue reservation.
